# Genotoxic and epigenotoxic effects in mice exposed to concentrated ambient fine particulate matter (PM_2.5_) from São Paulo city, Brazil

**DOI:** 10.1186/s12989-018-0276-y

**Published:** 2018-10-19

**Authors:** Antonio Anax Falcão de Oliveira, Tiago Franco de Oliveira, Michelle Francini Dias, Marisa Helena Gennari Medeiros, Paolo Di Mascio, Mariana Veras, Miriam Lemos, Tania Marcourakis, Paulo Hilário Nascimento Saldiva, Ana Paula Melo Loureiro

**Affiliations:** 1 Departamento de Análises Clínicas e Toxicológicas, Faculdade de Ciências Farmacêuticas, Universidade de São Paulo, Av. Prof. Lineu Prestes 580, Bloco 13 B, São Paulo, CEP 05508-000 Brazil; 2 Departamento de Bioquímica, Instituto de Química, Universidade de São Paulo, Av. Prof. Lineu Prestes 748, São Paulo, CEP 05508-000 Brazil; 3 Laboratório de Poluição Atmosférica Experimental – LIM05, Hospital das Clínicas, Faculdade de Medicina, Universidade de São Paulo, Av. Dr. Arnaldo 455, São Paulo, CEP 01246903 Brazil; 4 Instituto de Estudos Avançados, Universidade de São Paulo, R. do Anfiteatro, 513, São Paulo, CEP 05508060 Brazil; 5 Present address: Departamento de Farmacociências, Universidade Federal de Ciências da Saúde de Porto Alegre, Rua Sarmento Leite 245, Porto Alegre, Rio Grande do Sul CEP 90050-170 Brazil

**Keywords:** Particulate matter, Oxidative stress, DNA adducts, DNA methylation

## Abstract

**Background:**

The Metropolitan Area of São Paulo has a unique composition of atmospheric pollutants, and positive correlations between exposure and the risk of diseases and mortality have been observed. Here we assessed the effects of ambient fine particulate matter (PM_2.5_) on genotoxic and global DNA methylation and hydroxymethylation changes, as well as the activities of antioxidant enzymes, in tissues of AJ mice exposed whole body to ambient air enriched in PM_2.5_, which was concentrated in a chamber near an avenue of intense traffic in São Paulo City, Brazil.

**Results:**

Mice exposed to concentrated ambient PM_2.5_ (1 h daily, 3 months) were compared to in situ ambient air exposed mice as the study control. The concentrated PM_2.5_ exposed group presented increased levels of the oxidized nucleoside 8-oxo-7,8-dihydro-2′-deoxyguanosine in lung and kidney DNA and increased levels of the etheno adducts 1,*N*^6^-etheno-2′-deoxyadenosine and 1,*N*^2^-etheno-2′-deoxyguanosine in kidney and liver DNA, respectively. Apart from the genotoxic effects, the exposure to PM_2.5_ led to decreased levels of the epigenetic mark 5-hydroxymethylcytosine (5-hmC) in lung and liver DNA. Changes in lung, liver, and erythrocyte antioxidant enzyme activities were also observed. Decreased glutathione reductase and increased superoxide dismutase (SOD) activities were observed in the lungs, while the liver presented increased glutathione S-transferase and decreased SOD activities. An increase in SOD activity was also observed in erythrocytes. These changes are consistent with the induction of local and systemic oxidative stress.

**Conclusions:**

Mice exposed daily to PM_2.5_ at a concentration that mimics 24-h exposure to the mean concentration found in ambient air presented, after 3 months, increased levels of DNA lesions related to the occurrence of oxidative stress in the lungs, liver, and kidney, in parallel to decreased global levels of 5-hmC in lung and liver DNA. Genetic and epigenetic alterations induced by pollutants may affect the genes committed to cell cycle control, apoptosis, and cell differentiation, increasing the chance of cancer development, which merits further investigation.

**Electronic supplementary material:**

The online version of this article (10.1186/s12989-018-0276-y) contains supplementary material, which is available to authorized users.

## Background

Epidemiologic studies conducted mostly in the United States and Europe have shown that cardiovascular diseases, respiratory diseases, and cancer of different tissues (e.g., lung, breast, skin, hematopoietic, bladder, kidney, larynx, and thyroid) are empirically associated with exposure to air pollution [1–12]. Nonetheless, the balance between polluting activities and public policies to control air pollution dictates specific air pollutant compositions in each region, whose effects on disease risk, particularly cancer, are far from being completely understood [9].

The atmospheric chemical composition of the Metropolitan Area of São Paulo is characterized by emissions of approximately 2000 industries with high pollution potential and a fleet of approximately 7 million vehicles, which are the main sources of air pollutants in a region inhabiting approximately 21 million individuals [12, 13]. Automotive fuels in use are gasohol (gasoline containing 20–25% ethanol), hydrated ethanol (95% ethanol), compressed natural gas, and diesel containing 5% biodiesel [12], creating a unique composition of atmospheric pollutants with increased levels of formaldehyde, acetaldehyde, and ethanol compared to those found in other countries [14, 15]. Policies to reduce pollutant emissions by vehicles and industries in São Paulo State have been implemented since the 1980s and a diminishing tendency of regulated air pollutants (CO, hydrocarbons, nitrogen oxides, PM_10_, and sulfur oxides) has been observed, except for ozone, even with the increase in vehicular fleet and fuel consumption [12]. However, the current levels in São Paulo are far from the recommended limits of the WHO [16], and high positive correlations between air pollution exposure and risk of cardiovascular disease, cancer, and mortality have been observed, as also shown in developed countries [7, 17–19].

The particulate matter (PM) fraction of air pollution includes solid and liquid particles of aerodynamic diameters ranging from 5 nm to 100 μm, originating from fuel combustion, biomass burning, and nucleation events based on vapor condensation (sulfuric acid, nitric acid, organic matter) in the atmosphere [9, 12, 18]. Coarse PM, with a 2.5 to 10 μm aerodynamic diameter (PM_10_), are retained in the upper airways, while fine and ultrafine PM, with aerodynamic diameters in the 0.1–2.5 μm (PM_2.5_) range and smaller than 0.1 μm (PM_0.1_), respectively, can penetrate into the terminal portion of bronchi and alveoli [4, 7, 18], where they can elicit inflammatory reactions that increase the risk of diseases [2, 20, 21]. In addition to the lung absorption of particle compounds, airway mucociliary clearance results in gastrointestinal tract exposure to swallowed particles, which may trigger systemic effects [22]. Fine particles are important carriers of organic and inorganic chemicals, such as polycyclic aromatic hydrocarbons (PAHs), nitro-PAHs, aldehydes, ketones, carboxylic acids, quinolines, metals, and water-soluble ions, which may lead to harmful effects, such as inflammation, oxidative stress, mutations and cancer [10, 23–25]. Carcinogenic PAHs, such as benzo[*a*]pyrene, can be metabolized to reactive intermediates able to damage biomolecules, including the DNA [26, 27]. Diverse types of genome damage (chromosomal aberrations, DNA strand breaks, sister chromatid exchange, DNA adducts, micronuclei) and mutations have been shown as consequences of exposure to benzo[*a*]pyrene [26, 27]. The increase in the generation of reactive oxygen species (ROS) induced by PAHs may also contribute to the observed effects [28]. Based on strong evidence from epidemiological and experimental studies, the International Agency for Research on Cancer (IARC), in 2013, classified outdoor air pollution and airborne particulate matter as carcinogenic to humans (Group 1) [10].

**Fig. 1:**
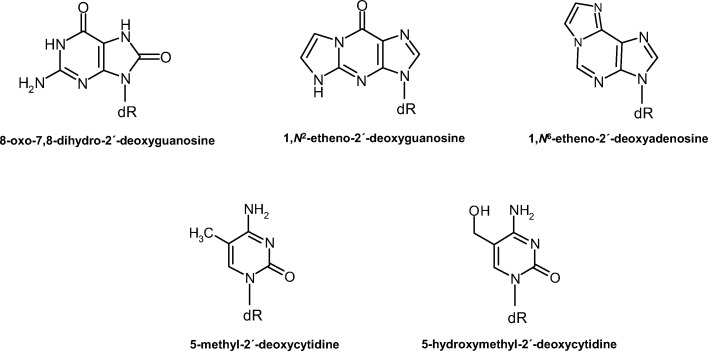
Chemical structures of the DNA lesions and epigenetic marks quantified in the present study. dR = 2′-deoxyribose

Among the useful biomarkers for the assessment of chemical carcinogenesis pathways, DNA adducts and oxidized nucleobases or nucleosides provide information on early events triggered by exposure, such as the bioactivation of xenobiotics and oxidative stress, while DNA methylation changes depict early biological effects that may evolve to cancer [5]. Accordingly, the levels of the oxidized base 8-oxo-7,8-dihydro-guanine (8-oxoGua) or the deoxyribonucleoside 8-oxo-7,8-dihydro-2′-deoxyguanosine (8-oxodGuo, Fig. 1) are consistently increased in the mononuclear blood cells and urine of subjects exposed to ambient air pollution [5]. Regarding cancer risk, lung 8-oxodGuo levels were correlated with the induction of lung tumors in mice exposed to diesel exhaust particles (DEPs) [29], while in humans the urinary excretion of 8-oxodGuo was associated with the risk of lung cancer [30] and the incidence of colorectal cancer and benign adenoma [31].

In addition to direct DNA oxidation, oxidative stress may induce the formation of mutagenic exocyclic DNA adducts, such as propano, etheno, and malonaldehyde adducts, via reactive carbonyls resulting from lipid peroxidation [32]. The potential role of these lesions as biomarkers of oxidative stress and in cancer etiology has been determined since their quantification in vivo became feasible by using highly sensitive and selective methods [33–43]. Interestingly, the etheno adducts 1,*N*^2^-etheno-2′-deoxyguanosine (1,*N*^2^-εdGuo, Fig. 1) and 1,*N*^6^-etheno-2′-deoxyadenosine (1,*N*^6^-εdAdo, Fig. 1) were the most increased (3- to 4-fold) DNA lesions among others investigated (DNA oxidation and deamination products) in spleen, liver and kidney DNA samples from the nitric oxide overproduction SJL mouse model of inflammation [37]. Despite their potential use as biomarkers in the pathophysiology of inflammation [36, 37], the assessment of these etheno adducts under conditions of PM exposure has only been addressed by a few studies focusing on the effects of wood smoke and ambient PM resulting from wood stoves [44, 45]. Regardless of the inflammatory response [20, 21, 46–48], there are no studies on the effects of urban air PM exposure on the tissue levels of DNA etheno adducts.

Current research has uncovered epigenetic changes associated with PM exposure, which may play a role in cardiovascular disease and cancer development [47, 49–52]. The current best characterized epigenetic mark is DNA 5-methylcytosine (5-mC, Fig. 1), maintained by DNA methyltransferases (DNMTs) at approximately 4% of the cytosine levels in adult human DNA, with important roles in development, genomic imprinting, silencing of transposable elements, and regulation of gene expression [50, 53–56]. While DNMTs catalyze the C-5 methylation of cytosine bases pertaining to CpGs sequences, the Ten-Eleven-Translocation (TET) methylcytosine hydroxylases are involved in the oxidation of 5-mC to 5-hydroxymethylcytosine (5-hmC, Fig. 1), which is considered the first step in the demethylation process [57].

Studies on global DNA methylation in the whole blood of humans have been associated with a hypomethylation effect of PM exposure [47, 49, 51, 58–62], whereas the outcomes of PM exposure on the global DNA methylation of other target tissues have been studied in mice sperm, rat lung, human placenta, and human buccal cells [63–67]. Increased levels of 5-hmC were detected in blood samples from humans exposed to ambient PM_10_ [68], but human bronchial epithelial cells (HBECs) exposed to DEPs (5 mg/cm^2^, 24 h) in vitro presented decreased 5-hmC levels [69]. Additionally, the increase in human exposure to PM_2.5_ or PM_10_ was associated with a decrease of 5-hmC levels in buccal cells [65].

To better understand the toxicity pathways of fine particulate matter, considering that studies focusing on the genotoxic and epigenotoxic effects of ambient air pollution in South America are scarce [5, 8], we used HPLC-ESI-MS/MS methods to evaluate the effects of ambient PM_2.5_ on genotoxic (8-oxodGuo, 1,*N*^2^-εdGuo and 1,*N*^6^-εdAdo lesions) and global DNA 5-mC and 5-hmC changes in different tissues (lung, liver and kidney) of AJ mice. The animals were exposed whole body to ambient air enriched in PM_2.5_ and compared to in situ ambient air exposed mice as the study control. The fine particulate matter was concentrated in a chamber placed near an avenue of intense traffic in São Paulo City, Brazil.

## Methods

### Chemicals and enzymes

All the chemicals employed here were of the highest purity grade commercially available. Chromatography grade acetonitrile and methanol, isopropyl alcohol, chloroform, hydrochloric acid, ethanol, magnesium chloride, and tris(hydroxymethyl)-aminomethane were obtained from Carlo Erba Reagents (Milan, Italy). Sodium hydroxide, potassium phosphate, and ammonium acetate were acquired from Merck (Darmstadt, Germany). DNA extraction solutions were obtained from QIAGEN (Valencia, CA). DNase I was acquired from Bio Basic Inc. (Ontario, Canada). [^15^N_5_]-2′-deoxyguanosine and [^15^N_5_]-2′-deoxyadenosine were provided by Cambridge Isotope Laboratories (Andover, MA). All the other reagents were obtained from Sigma-Aldrich Co. (St. Louis, MO). The catalogue numbers of the reagents are described in Additional file 1: Table S1. Water was purified in a Milli-Q system (Millipore, Bedford, MA).

### Adduct standards

1,*N*^2^-Etheno-2′-deoxyguanosine (1,*N*^2^-εdGuo) and the isotopic standards [^15^N_5_]1,*N*^2^-εdGuo, [^15^N_5_]1,*N*^6^-εdAdo and [^15^N_5_]8-oxodGuo were prepared as described [35, 70]. Their identities were confirmed by their spectral properties, as follows. 1,*N*^2^-εdGuo: UV, *λ*_max_ = 285 nm, ε = 16,785 M^− 1^ cm^− 1^ [71], pH 7.0; positive ESI-MS: *m/z* 292 [M + H]^+^, *m/z* 176 [M – 2′-deoxyribose + H]^+^. [^15^N_5_]1,*N*^2^-εdGuo: positive ESI-MS: *m/z* 297 [M + H]^+^, *m/z* 181 [M – 2′-deoxyribose + H]^+^. [^15^N_5_]1,*N*^6^-εdAdo: UV, *λ*_max_ = 260 nm, ε = 10,300 M^− 1^ cm^− 1^ [72], pH 7.0; positive ESI-MS: *m/z* 281 [M + H]^+^, *m/z* 165 [M – 2′-deoxyribose + H]^+^. [^15^N_5_]8-oxodGuo: UV, *λ*_max_ = 294 nm, ε = 9700 M^− 1^ cm^− 1^ [73] in water; positive ESI-MS: *m/z* 289 [M + H]^+^, *m/z* 173 [M – 2′-deoxyribose + H]^+^.

### 5-mC standard

5-methyl-2′-deoxycytidine (5-mC) standard was obtained by HPLC purification from a solution of commercial dCyd (1 mg/mL). Its spectral properties are as follows: UV in H_2_O, *λ*_max_ 277 nm, ε = 8500 M^− 1^ cm^− 1^ [74]; positive ESI-MS: *m/z* 242 [M + H]^+^, *m/z* 126 [M – 2′-deoxyribose + H]^+^. The standard was used for quantification of 5-mC and 5-hmC, but the retention time of 5-hmC was defined using a sample of calf thymus DNA presenting a main peak with *m*/*z* 258 [M + H]^+^ → *m*/*z* 142 [M - 2′-deoxyribose + H]^+^.

### Experimental groups

Four week old male AJ mice, specific pathogen free, were obtained from the Breeding Center of Laboratory Animals of Fundação Oswaldo Cruz (FIOCRUZ), Rio de Janeiro, Brazil, and were treated accordingly to the Ethics Committee of the Faculty of Medicine, University of São Paulo (protocol n^⍛^ 1310/09). The animals were submitted to a controlled exposure system in a Harvard Ambient Fine-Particle Concentrator designed to concentrate PM_2.5_ up to 30 times. It works separating particles from gases by particle inertia using virtual impaction [75]. It has two chambers, one receiving the air concentrated with particles and the other receiving the ambient air. Two experimental groups were considered, eight animals each: one group breathed concentrated PM_2.5_ and the other group breathed ambient air. They were exposed 1 h/day in the afternoon, 5 days/week, along 3 months (September to November). Between exposures, animals were maintained in plastic cages, at a controlled temperature, in a 12-h light-dark cycle; they also received clean air (HEPA air), food (CR-1 Nuvilab, Colombo, PR, Brazil) and water ad libitum. Immediately after the last exposure, they were anesthetized by i.p. injection of xylazine and ketamine (87.5 mg/kg ketamine and 12.5 mg/kg xylazine). Blood samples were collected, centrifuged (2000 *g*), and the red blood cells separated for antioxidant enzyme activity assessment. Animals were euthanized by exsanguination. Lung, liver, and kidney were immediately collected, frozen in liquid nitrogen and kept under -80 °C until processing.

### Site of exposure

The exposure site was the same described by Akinaga and colleagues [76], located in an area of the Medical School of University of São Paulo, 20 m from the roadside and 150 m from a busy traffic crossroad. The Cerqueira César monitoring station of the São Paulo State Environmental Agency (CETESB, São Paulo, Brazil) is located 160 m from the exposure site. The region is impacted mainly by the emissions of light duty gasohol / hydrated ethanol and heavy duty diesel vehicles.

### Exposure chambers

Animals were maintained in special cages that allowed homogeneous distribution of air. Both experimental groups were exposed at the same time and conditions of temperature and pressure. Animals in the 30 times concentrator chamber were exposed daily to PM_2.5_ (5 days/week, 3 months), constantly monitored by a nephelometer. The calculated PM_2.5_ concentration (mean ± SD) for the entire period of exposure was 682 ± 532 μg/m^3^. Twice a week, samples of the PM_2.5_ were collected onto polycarbonate filters for elemental analysis [77]. For determination of specific polycyclic aromatic hydrocarbons in PM_2.5_, we used a Hi-vol particle sampler to collect these particles on quartz filters. Analyses of these filters were conducted according to Magalhães and coworkers [78].

### Antioxidant enzyme activities

Enzymatic activities of glutathione peroxidase (GPx), glutathione reductase (GR), glutathione S-transferase (GST), and superoxide dismutase (SOD) were determined by spectrophotometric method in lung, liver, kidney, and erythrocytes. Catalase activity (CAT) was evaluated only in erythrocytes. GPx activity was performed using the procedure described by Flohé and Günzler [79]. Tert-butyl hydroperoxide was used as substrate and the formation of oxidized glutathione (GSSG) was indirectly monitored spectrophotometrically through NADPH consumption at 340 nm during 5 min. GR activity was assayed according to Carlberg and Mannervik [80]. The reduction of GSSG to GSH was measured through NADPH consumption and monitored spectrophotometrically at 37 °C for 10 min at 340 nm. GST activity assay was conducted measuring the conjugation of 1-chloro-2,4-dinitrobenzene (CDNB) with reduced glutathione, according to Habig [81]. The formation of the complex was monitored spectrophotometrically at 25 °C for 5 min at 340 nm. Superoxide dismutase (SOD) activity was measured based on McCord & Fridovich, and Flohé & Ötting methods that use the system hypoxanthine-xanthine as a superoxide anion donor [82, 83]. The O_2_^•–^ production is coupled to the reduction of ferricytochrome C, which is followed spectrophotometrically, allowing for quantitative measurement. SOD in samples converts superoxide free radical to H_2_O_2_ and O_2_, therefore slowing the rate of ferricytochrome C reduction. During the assay, the absorbance was detected in a spectrophotometer at 550 nm, 25 °C. SOD, GPx, GR and GST assays were performed in spectrophotometer Power Wave × 340 (Bio-Tek Instruments INC, software KC4 v3.0). Catalase activity was evaluated measuring the consumption of hydrogen peroxide (H_2_O_2_) [84]. The decrease in absorbance was monitored at 25 °C for 30 s at 240 nm in a spectrophotometer (Biochrom Libra S12). All enzyme activities in lung, liver and kidney are expressed as U/μg of protein. The enzyme activities conducted in erythrocytes were corrected by hemoglobin content and expressed as U/g of hemoglobin. Protein and hemoglobin contents were determined by Bradford and Doles® reagents, respectively. All enzymatic assays were conducted in triplicate.

### DNA extraction

DNA samples from lung, liver, and kidney were extracted using a QIAGEN kit according to manufacturer’s instructions for 1 g of animal tissue, maintaining the correct proportions. Briefly, tissue samples (with average weight of 1.0 g) were homogenized with 10 mL of the cell lysis solution (QIAGEN, Cat. No. 158908) plus 0.5 mM deferoxamine. The obtained homogenates were added to 150 μL of 20 mg/mL proteinase K solution. The samples were homogenized and remained at room temperature overnight. After this period, 40 μL of ribonuclease A (15 mg/mL) were added and kept 2 h at room temperature. The proteins were precipitated by adding 5 mL of protein precipitation solution (QIAGEN, Cat. No. 158912) and centrifuged at 2000 *g* for 10 min. The supernatants were transferred to tubes containing 10 mL of cold isopropanol. The precipitated DNA was collected into tubes containing 4 mL of 10 mM Tris buffer, 1 mM deferoxamine, pH 7.0, and extracted three times with 4 mL of a chloroform solution containing 4% of isoamyl alcohol. The DNA was again precipitated by adding 8 mL of absolute ethanol and 0.4 mL of a 5 M NaCl solution, and washed twice with 3 mL of 70% ethanol. After air drying, the samples were suspended in 200 μL of 0.1 mM deferoxamine solution and stored at -20 °C. The DNA concentration was determined by measuring the absorbance at 260 nm and its purity was established based on the 260/280 nm absorbance ratio.

### DNA enzymatic hydrolysis

For analyses of etheno adducts in liver, aliquots containing 150 μg of DNA were transferred to a final volume of 200 μL of deionized water. 7.5 μL of 200 mM Tris/MgCl_2_ buffer (pH 7.4), 1.4 μL of the internal standard solution containing [^15^N_5_]1,*N*^*6*^-εdAdo and [^15^N_5_]1,*N*^*2*^-εdGuo (250 fmol/μL), and 6 μL (15 units) of deoxyribonuclease I (Bio Basic Inc., Ontario, Canada) were added. The samples were incubated at 37 °C, 90 rpm for 1 h. Then, 6 μL (0.006 units) of phosphodiesterase I from *Crotalus atrox* (Sigma Aldrich, St. Louis, MO, USA) and 7.5 μL (15 units) of alkaline phosphatase from bovine intestinal mucosa (Sigma Aldrich, St. Louis, USA) were added, incubating again at 37 °C, 90 rpm for 1 h. Finally, samples were centrifuged at 14,000 *g* for 10 min. Aliquots of 10 μL were withdrawn for quantification of deoxynucleosides (dAdo, dGuo) by HPLC/PDA. The residual volume of sample was submitted to solid phase extraction, as described below.

Analyses of etheno adducts in kidney and lung were performed using 100 μg of DNA, maintaining the correct proportions of enzymes and the other reagents.

The same procedure was used for quantification of 8-oxodGuo in liver, lung and kidney, using 80 μg of DNA and 1000 fmol of the internal standard [^15^N_5_]8-oxodGuo in the injection volume.

Samples containing 12 μg DNA and 3000 fmol of [^15^N_5_]1,*N*^*6*^-εdAdo were also hydrolyzed for analyses of 5-mC and 5-hmC, using the same procedure adjusted to a final volume of 60 μL. The hydrolyzed samples were, then, added to 140 μL of acetonitrile, vortexed for 20 s, centrifuged at 9300 *g* for 10 min, and aliquots of 20 μL were injected into the HPLC-ESI-MS/MS system described below.

### Solid phase extraction

Samples for the analyses of etheno adducts were pre-purified by solid phase extraction, using SPE-C18 cartridges (30 mg/mL, 33 μm, 1 mL, Strata-X, Phenomenex, Torrance, CA, Cat. No. 8B-S100-TAK). This step was not performed for quantification of 8-oxodGuo. The cartridges were loaded in the following sequence: 100% methanol, deionized water, hydrolyzed DNA sample, deionized water, 10% methanol, 15% methanol, and 100% methanol. The last elution fraction containing the adducts of interest was collected. Samples were then vacuum dried and resuspended in 83.1 μL of MiliQ water immediately prior to the HPLC-ESI-MS/MS analysis, to obtain 200 fmol of internal standards in 50 μL of each sample.

### Quantification of DNA lesions, 5-mC and 5-hmC

The levels of 1,*N*^*6*^-εdAdo, 1,*N*^*2*^-εdGuo, 8-oxodGuo, 5-mC and 5-hmC in DNA samples were assessed by HPLC-ESI-MS/MS. The analytical system consisted of an Agilent 1200 series HPLC (Wilmington, DE, USA) equipped with a binary pump (Agilent 1200 G1312B), an isocratic pump (Agilent 1200 G1310A), a column oven (Agilent 1200 G1316B), a diode array detector (Agilent 1200 DAD G1315C), and an auto sampler (G1367C Agilent 1200) interfaced with a Linear Quadrupole Ion Trap mass spectrometer, Model 4000 QTRAP (Applied Biosystems/MDS Sciex Instruments, Foster City). The ESI-MS/MS parameters were set in the positive ion mode as described in Additional file 1: Table S2.

Analyses were carried out with multiple reaction monitoring (MRM) by using the following fragmentations: *m*/*z* 276 [M + H]^+^ → *m*/*z* 160 [M - 2′-deoxyribose + H]^+^ and *m*/*z* 281 [M + H]^+^ → *m*/*z* 165 [M - 2′-deoxyribose + H]^+^ for detection of 1,*N*^6^-εdAdo and respective internal standard [^15^N_5_]1,*N*^6^-εdAdo; *m*/*z* 292 [M + H]^+^ → *m*/*z* 176 [M - 2′-deoxyribose + H]^+^ and *m*/*z* 297 [M + H]^+^ → *m*/*z* 181 [M - 2′-deoxyribose + H]^+^ for detection of 1,*N*^2^-εdGuo and respective internal standard [^15^N_5_]1,*N*^2^-εdGuo; *m/z* 284 [M + H]^+^ → *m/z* 168 [M - 2′-deoxyribose + H]^+^ and *m/z* 289 [M + H]^+^ → *m/z* 173 [M - 2′-deoxyribose + H]^+^ for detection of 8-oxodGuo and respective internal standard [^15^N_5_]8-oxodGuo; *m*/*z* 242 [M + H]^+^ → *m*/*z* 126 [M - 2′-deoxyribose + H]^+^ for detection of 5-mC; *m*/*z* 258 [M + H]^+^ → *m*/*z* 142 [M - 2′-deoxyribose + H]^+^ for detection of 5-hmC; *m*/*z* 228 [M + H]^+^ → *m*/*z* 112 [M - 2′-deoxyribose + H]^+^ for detection of dCyd.

The calibration curves were constructed at the intervals of 367 to 5875 fmol of 8-oxodGuo, with a fixed amount of [^15^N_5_]8-oxodGuo (1000 fmol); 1 to 40 fmol of 1,*N*^6^-εdAdo and 1,*N*^2^-εdGuo, with fixed amounts of [^15^N_5_]1,*N*^6^-εdAdo and [^15^N_5_]1,*N*^2^-εdGuo (200 fmol); and 150 to 1500 pmol of dCyd, 5 to 500 fmol of 5-mC for quantification of 5-hmC, and 5 to 80 pmol of 5-mC for quantification of 5-mC, with a fixed amount of [^15^N_5_]1,*N*^6^-εdAdo (300 fmol). Data were acquired and processed using Analyst software 1.4 (Applied Biosystems/MDS Sciex). The molar fractions 8-oxodGuo/dGuo, 1,*N*^6^-εdAdo/dAdo, 1,*N*^2^-εdGuo/dGuo, 5-mC/(5-mC + 5-hmC + dCyd), 5-hmC/(5-mC + 5-hmC + dCyd) present in each DNA sample were determined. The following chromatography conditions were used for the analyses. All solvents used were previously filtered and degassed.

8-oxodGuo analysis: A 50 × 2.0 mm i.d., 2.5 μm, Luna C18(2)-HST column (Phenomenex, Torrance, CA) with a C18(2) security guard cartridge, 4.0 × 3.0 mm i.d. (Phenomenex, Torrance, CA) was eluted with a gradient of 0.1% formic acid (solvent A) and methanol containing 0.1% formic acid (solvent B) at a flow rate of 150 μL/min and 25 °C, as follows: from 0 to 25 min, 0–15% of solvent B; 25 to 28 min, 15–80% of solvent B; 28 to 31 min, 80% of solvent B; 31 to 33 min, 80–0% of solvent B; 33 to 46 min, 0% of solvent B. The first 16 min of eluent was directed to waste and the 16–32 min fraction was diverted to a second column (150 × 2.0 mm i.d., 3.0 μm, Luna C18(2)) connected to the ESI source and conditioned by a third isocratic pump with a solution of 15% methanol in water containing 0.1% formic acid (150 μL/min) (Additional file 1: Figure S1). The lesion 8-oxodGuo eluted from the second column at approximately 36 min.

Etheno adducts analyses: A 150 × 2.0 mm i.d., 3.0 μm, Luna C18(2) column (Phenomenex, Torrance, CA) with a C18(2) security guard cartridge, 4.0 × 3.0 mm i.d. (Phenomenex, Torrance, CA) was eluted with a gradient of 5 mM ammonium acetate, pH 6.6 (solvent A) and acetonitrile (solvent B) at a flow rate of 130 μL/min and 25 °C, as follows: from 0 to 10 min, 0% of solvent B; 10 to 39 min, 0–20% of solvent B; 39 to 41 min, 20–75% of solvent B; 41 to 46 min, 75% of solvent B; 46 to 47 min, 75–0% of solvent B; 47 to 60 min, 0% of solvent B. The first 15 min of eluent was directed to waste and the 15–18 min fraction was diverted to the ESI source.

5-mC and 5-hmC analyses: A 150 × 4.6 mm i.d., 5 μm, *Syncronis* HILIC(2) column (Thermo Scientific, USA), with a HILIC security guard cartridge, 4.0 × 3.0 mm i.d. (Thermo Scientific, USA), was eluted with a gradient of acetonitrile (solvent A) and 5 mM ammonium acetate, pH 8.2 (solvent B) at a flow rate of 300 μL/min and 35 °C, as follows: from 0 to 35 min, 0–40% of solvent B; 35 to 36 min, 40–0% of solvent B; 36 to 56 min, 0% of solvent B. The first 16 min of eluent was directed to waste and the 16–27 min fraction was diverted to the ESI source.

### Method validation for 8-oxodGuo quantification in DNA samples

Method accuracy and precision were determined by adding varying amounts of 8-oxodGuo (367, 734, 1469, and 2204 fmol) and a fixed amount of [^15^N_5_]8-oxodGuo (1000 fmol) to 100 μg of calf thymus DNA and carrying out the enzymatic hydrolysis and analysis. Samples were processed in quadruplicate in two different days. The limit of quantitation (LOQ) was estimated from the lowest amount of 8-oxodGuo injected on column, with S/N = 10.

### Method validation for 1,*N*^6^-εdAdo and 1,*N*^2^-εdGuo quantification in DNA samples

Varying amounts of 1,*N*^6^-εdAdo (1, 5, 10, and 20 fmol in the injection volume) and 1,*N*^2^-εdGuo (1, 5, 10, and 20 fmol in the injection volume) and fixed amounts of [^15^N_5_]1,*N*^6^-εdAdo and [^15^N_5_]1,*N*^2^-εdGuo (200 fmol in the injection volume) were added to 100 μg of calf thymus DNA and the analyses were carried out. Samples were processed in quadruplicate in two different days for method accuracy and precision assessment. Recovery was calculated by adding the internal standards [^15^N_5_]1,*N*^6^-εdAdo and [^15^N_5_]1,*N*^2^-εdGuo (200 fmol) to 100 μg of calf thymus DNA before and after solid phase extraction, carrying out the analyses as described above. The DNA used for recovery calculation was contaminated with 7.5 fmol of 1,*N*^6^-εdAdo and 20 fmol of 1,*N*^2^-εdGuo in the beginning of the process. The limit of detection (LOD) was estimated from the lowest amount of adducts added to the calf thymus DNA sample.

### Normal 2′-deoxynucleosides quantification in DNA samples used for analyses of 8-oxodGuo, 1,*N*^6^-εdAdo and 1,*N*^2^-εdGuo

The quantification of normal 2′-deoxynucleosides was carried out with a Shimadzu (Kyoto, Japan) HPLC system equipped with two LC-20AT pumps, a photo diode array detector (PDA-20AV), an auto-injector (Proeminence SIL-20 AC), and a column oven (CTO-10AS/VP) controlled by a CBM-20A communication module and the software LC-Solution. Elution system was as follows: a 250 mm × 4.6 mm i.d., 5 μm, Luna C18(2) column (Phenomenex, Torrance, CA) attached to a C18(2) guard column (4,0 × 3.0 mm i.d., 4 μm, Phenomenex, Torrance, CA), eluted with a gradient of 0.1% formic acid and CH_3_OH (from 0 to 25 min, 0 to 18% CH_3_OH; from 25 to 27 min, 18 to 0% CH_3_OH; from 27 to 37 min, 0% CH_3_OH) at a flow rate of 1 mL/min and 30 °C. The PDA detector was set at 260 nm. Calibration curves were constructed at intervals of 0.05–1 nmol for dGuo and dAdo.

### Statistics

Data were expressed as average ± SEM or average ± SD, as indicated in the text and tables. Means between the two groups (ambient air and PM_2.5_) were compared using t test for 8-oxodGuo, 1,*N*^2^-εdGuo, 1,*N*^6^-εdAdo, 5-mC and antioxidant enzymes activities, or Mann-Whitney test for 5-hmC. Results were considered statistically significant when *P* value was less than 0.05. Normality of the samples was checked by the Kolmogorov-Smirnov test and homogeneity of variances with the Levene test. The statistical analyses were conducted using GraphPad Prism version 6 for Windows (GraphPad Software, San Diego California USA).

## Results

### Exposure assessment

The elemental characterization of PM_2.5_ is depicted in Table 1. Table 2 presents the mean concentrations of specific polycyclic aromatic hydrocarbons detected in PM_2.5_ collected near the site of exposure.

**Table 1:** Elements (ng/m^3^) detected in PM_2.5_ (μg/m^3^)

	Minimum	Q1	Median	Q3	Maximum	Mean	SD	SEM
PM_2.5_	65.2	222	574	1047	2094	682	532	95.5
Na	0.00	10.68	432.95	613.42	3774.50	525.64	711.31	125.74
Mg	0.00	0.00	44.13	302.58	6061.46	363.25	1059.70	187.33
Al	289.82	1130.75	1785.09	4219.14	65,846.50	5610.86	11,559.93	2043.53
Si	93.81	827.61	1840.58	4954.59	83,737.34	6489.89	14,685.73	2596.10
P	0.00	39.55	219.26	512.15	8645.44	584.46	1492.15	263.78
S	573.18	2935.17	7074.47	12,747.56	27,164.33	9374.97	8051.74	1423.36
Cl	0.00	75.99	433.59	932.43	4994.57	772.92	1038.12	183.52
K	62.70	778.64	1488.44	3005.62	35,421.57	3458.71	6403.12	1131.92
Ca	44.18	447.99	1233.81	2473.66	40,539.07	3267.85	7148.36	1263.66
Ti	8.70	54.86	151.17	564.01	6261.38	641.99	1429.13	252.64
V	0.00	0.73	6.43	14.18	71.68	11.50	16.50	2.92
Cr	0.00	0.00	0.00	13.09	198.64	14.21	35.27	6.23
Mn	6.21	47.19	90.59	121.04	1432.81	157.48	255.25	45.12
Fe	5.74	1094.30	2042.59	4479.96	65,800.18	5304.88	11,441.17	2022.53
Ni	0.00	0.00	2.96	6.74	81.34	7.99	15.40	2.72
Cu	0.00	23.14	54.24	102.69	868.51	99.66	155.25	27.44
Zn	0.00	146.45	300.87	509.58	3411.48	500.06	703.91	124.43
Se	0.00	0.00	0.00	0.16	10.27	1.24	2.69	0.48
Br	0.00	10.55	15.17	45.03	223.70	36.15	45.36	8.02
Rh	0.00	0.00	0.00	80.53	391.62	51.97	91.40	16.42
Pb	0.00	9.68	31.84	36.13	207.06	37.88	45.23	10.66

**Table 2:** Polycyclic aromatic hydrocarbons in PM_2.5_ collected at the site of exposure

	Filters (*n*)	Mean ± SEM (range) ng/m^3^
Pyrene	6	1.66 ± 0.42 (3.6–9.0)
Benz[*e*]acephenantrylene	6	6.81 ± 1.88 (2.44–13.97)
Benzo[*k*]fluoranthene	8	1.42 ± 0.37 (0.44–3.17)
Benzo[*a*]pyrene	7	0.72 ± 0.14 (0.27–1.083)

### Antioxidant enzyme activities

Mice exposed to concentrated PM_2.5_ presented changes in lung, liver, and erythrocyte antioxidant enzyme activities, as shown in Table 3. Statistically significant decreased GR and statistically significant increased SOD activities were observed in the lungs, while the liver presented statistically significant increased GST and statistically significant decreased SOD activities. A statistically significant increase in SOD activity was also observed in erythrocytes.

**Table 3:** Enzymatic activities of glutathione peroxidase (GPx), glutathione reductase (GR), glutathione S-transferase (GST), superoxide dismutase (SOD), and catalase (CAT) in lung, liver, kidney (U/μg protein), and erythrocytes (U/g hemoglobin)

	Ambient Air	PM_2.5_	Number	*P* value
	Average ± SEM (U/μg protein or U/g hemoglobin)	Average ± SEM (U/μg protein or U/g hemoglobin)		
Lung				
GPx	2.832 ± 0.083	2.967 ± 0.114	4	NS
GR	1.454 ± 0.047	1.142 ± 0.074	4	0.02
GST	12.96 ± 0.600	12.96 ± 0.480	4	NS
SOD	133,436 ± 5858	190,269 ± 13,951	4	0.02
Liver				
GPx	9.900 ± 0.110	10.11 ± 0.150	5	NS
GR	1.390 ± 0.040	1.350 ± 0.060	5	NS
GST	31.82 ± 1.040	35.25 ± 0.700	5	0.02
SOD	296,609 ± 37,767	142,425 ± 21,046	5	<0.01
Kidney				
GPx	6.540 ± 0.090	6.170 ± 0.230	5	NS
GR	3.110 ± 0.080	2.910 ± 0.140	5	NS
GST	13.37 ± 0.240	13.020 ± 0.570	5	NS
SOD	114,203 ± 7149	109,789 ± 17,576	5	NS
Erythrocytes				
GPx	2175 ± 72.00	2292 ± 63.00	6	NS
GR	103.8 ± 4.500	97.98 ± 3.070	5	NS
GST	168.0 ± 7.900	173.8 ± 3.800	4	NS
SOD	151,304 ± 14,350	216,638 ± 4807	4	<0.01
CAT	110,883 ± 5300	113,442 ± 3196	6	NS

*NS* Not Significant

### Genotoxic effects of PM_2.5_ in the lungs, liver, and kidneys

**Fig. 2:**
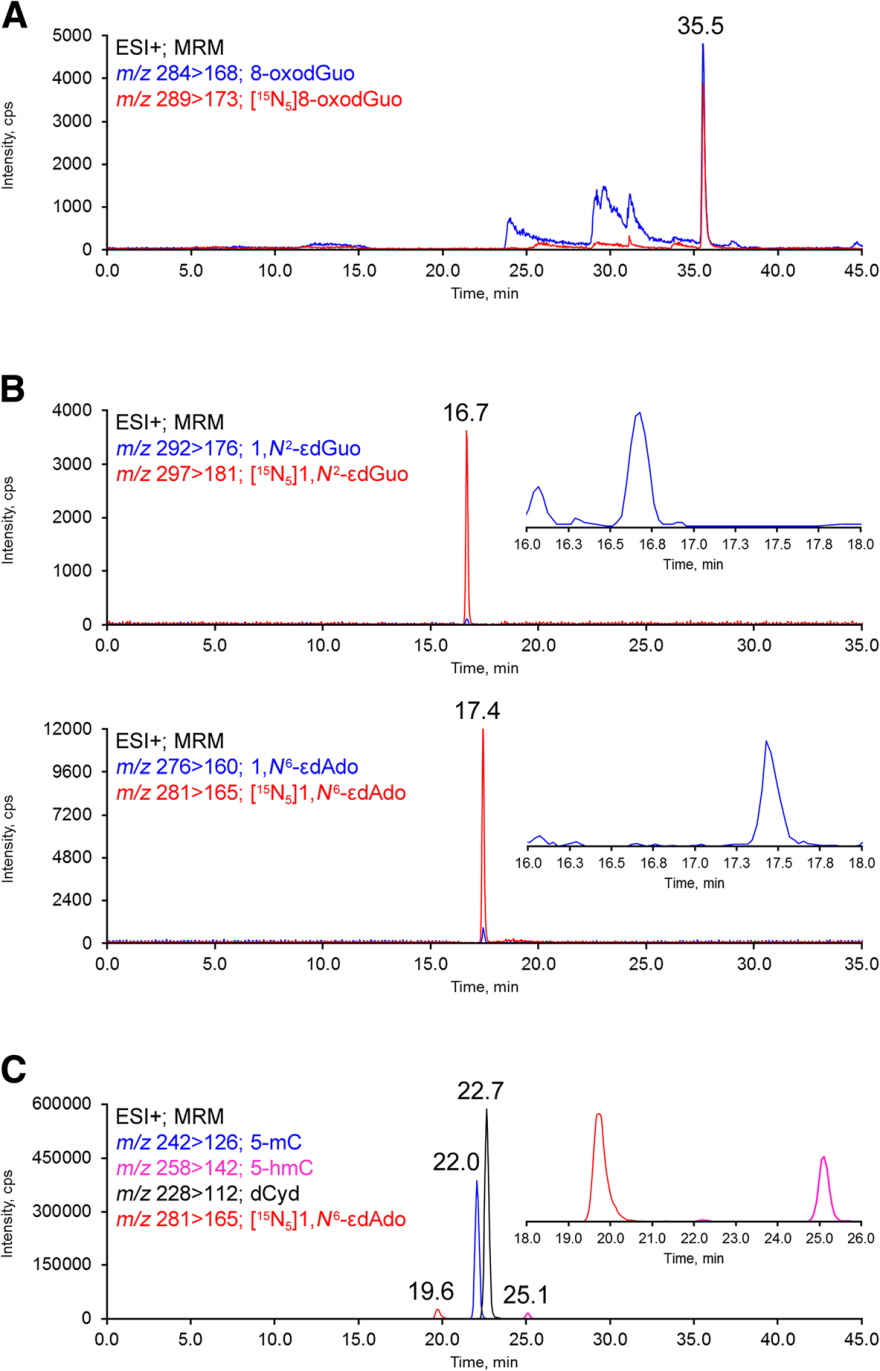
Representative chromatograms of DNA samples obtained by HPLC-ESI-MS/MS showing **a** 8-oxo-7,8-dihydro-2′-deoxyguanosine (8-oxodGuo); **b** 1,*N*^2^-etheno-2′-deoxyguanosine (1,*N*^2^-εdGuo) and 1,*N*^6^-etheno-2′-deoxyadenosine (1,*N*^6^-εdAdo); **c** 5-methyl-2′-deoxycytidine (5-mC), 5-hydroxymethyl-2′-deoxycytidine (5-hmC), and 2′-deoxycytidine (dCyd). The analyses were performed with multiple reaction monitoring (MRM) by using the fragmentations specified in the images for each analyte. The internal standards are represented by the red traces

The genotoxic effects of PM_2.5_ were assessed by the quantification of 8-oxodGuo and the etheno adducts 1,*N*^2^-εdGuo and 1,*N*^6^-εdAdo in lung, liver, and kidney DNA samples. The levels of each lesion in each organ of mice exposed to PM_2.5_ concentrated 30 times compared to those exposed to ambient air are shown in Table 4. The levels of 8-oxodGuo were increased in the lung and kidney DNA of mice exposed to concentrated PM_2.5_, while 1,*N*^2^-εdGuo was preferentially formed in the liver DNA of mice exposed to concentrated PM_2.5_. A significant increase in 1,*N*^6^-εdAdo was observed in the kidneys of mice exposed to concentrated PM_2.5_. Representative chromatograms are shown in Fig. 2a and b.

**Table 4:** Levels of DNA lesions, 5-mC and 5-hmC in samples from AJ mice exposed to ambient air and to PM_2.5_ concentrated 30 times

	Ambient Air	PM_2.5_	Number	*P* value
	Average ± SEM	Average ± SEM		
Lung				
8-oxodGuo/10^8^ dGuo	2124 ± 56.96	2466 ± 93.10	6	0.01
1,*N*^2^-εdGuo/10^8^ dGuo	ND	ND	–	–
1,*N*^6^-εdAdo/10^8^ dAdo	1.41 ± 0.23	1.44 ± 0.13	7	NS
5-mC (%)	3.53 ± 0.18	3.84 ± 0.21	8	NS
5-hmC (%)	0.044 ± 0.006	0.029 ± 0.001	8	<0.01
Liver				
8-oxodGuo/10^8^ dGuo	2848 ± 183.5	2949 ± 223.8	6; 5	NS
1,*N*^2^-εdGuo/10^8^ dGuo	7.79 ± 2.49	24.94 ± 5.21	4	0.02
1,*N*^6^-εdAdo/10^8^ dAdo	2.82 ± 0.30	2.18 ± 0.25	6	NS
5-mC (%)	4.68 ± 0.11	4.36 ± 0.38	8; 6	NS
5-hmC (%)	0.060 ± 0.004	0.051 ± 0.002	8; 6	0.04
Kidney				
8-oxodGuo/10^8^ dGuo	1854 ± 87.13	2363 ± 157.0	6	0.02
1,*N*^2^-εdGuo/10^8^ dGuo	ND	ND	–	–
1,*N*^6^-εdAdo/10^8^ dAdo	1.09 ± 0.15	1.52 ± 0.12	7	0.04
5-mC (%)	4.84 ± 0.24	4.20 ± 0.37	8	NS
5-hmC (%)	0.048 ± 0.003	0.048 ± 0.003	8	NS

*NS* Not Significant, *ND* Not Detected

### Effects of PM_2.5_ on global 5-mC and 5-hmC in lung, liver, and kidney DNA

**Fig. 3:**
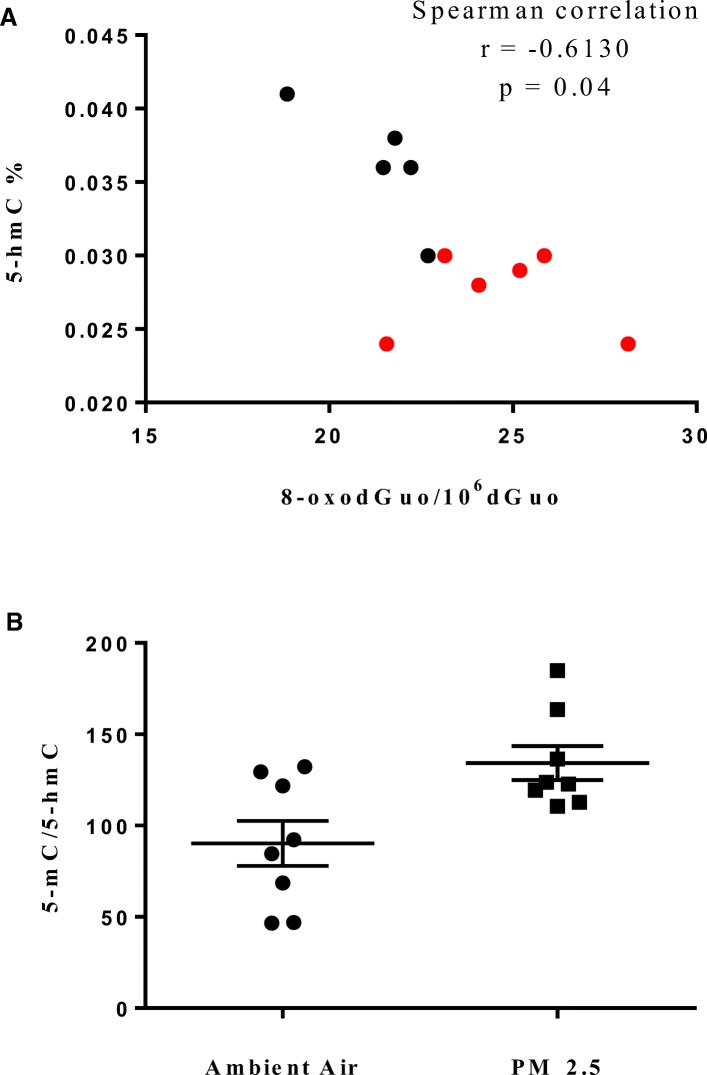
**a** Spearman correlation between 5-hmC and 8-oxodGuo in mice lung DNA (*N* = 11, five samples from ambient air are shown in black, and six samples from concentrated PM_2.5_ are shown in red); **b** Ratios between 5-mC and 5-hmC in mice lung DNA (Mean ± SEM, unpaired t test, *p* = 0.01)

Epigenetic effects of PM_2.5_ were evaluated by quantification of 5-mC and 5-hmC in lung, liver, and kidney DNA samples. The levels obtained for each organ from mice exposed to PM_2.5_ concentrated 30 times compared to those of mice exposed to ambient air are shown in Table 4. While exposure to concentrated PM_2.5_ did not lead to statistically significant changes in global 5-mC levels, the lung and liver samples of AJ mice exposed to concentrated PM_2.5_ presented lower levels of 5-hmC. An inverse correlation between 5-hmC and 8-oxodGuo was observed in the lung DNA (Fig. 3a). The ratios 5-mC/5-hmC in the lung DNA also differed significantly between the ambient air and PM_2.5_ groups (Fig. 3b). A representative chromatogram is shown in Fig. 2c.

### Method validation for 8-oxodGuo quantification in DNA samples

For 8-oxodGuo quantification, the system illustrated in Additional file 1: Figure S1 was used. A typical calibration curve in the 367–5875 fmol range, with a fixed amount of the internal standard [^15^N_5_]8-oxodGuo (1000 fmol), is presented in Additional file 1: Figure S2. The MS response was linear in the range measured. The method accuracy and precision are presented in Table 5. Good agreement was observed between added and detected amounts of 8-oxodGuo (86–104% accuracy). The intra-day precision was determined by calculating the coefficient of variation (%) for each concentration. The inter-day precision calculated for DNA aliquots supplemented with 367 fmol of 8-oxodGuo was 16.97%. The LOQ (S/N = 10) was 25 fmol for the standard on-column injection.

**Table 5:** Method accuracy and coefficient of variation (CV) for quantification of 8-oxodGuo, 1,*N*^2^-εdGuo and 1,*N*^6^-εdAdo in DNA

Basal level	Added	Detected	Detected(−)Basal	Accuracy	CV
Average ± SD (fmol)	fmol	Average ± SD (fmol)	Average (fmol)	%	%
8-oxodGuo					
373.00 ± 2.71	0	372.79 ± 50,60	–	–	13.57
373.98 ± 4.86	367	755.41 ± 107,92	381	103.93	14.29
374.84 ± 5.19	734	1069.57 ± 108,51	695	94.65	10.14
357.94 ± 15.05	1469	1671.67 ± 44,27	1314	89.43	2.65
371.07 ± 2.43	2204	2272.01 ± 40,20	1901	86.25	1.77
1,*N*^2^-εdGuo					
0.54 ± 0.01	0	0.54 ± 0.09	–	–	16.88
0.54 ± 0.01	1	1.47 ± 0.16	0.93	93.39	11.17
0.55 ± 0.01	5	5.30 ± 0.72	4.76	95.11	13.50
0.53 ± 0.01	10	10.60 ± 0.39	10.06	100.63	3.67
0.54 ± 0.01	20	20.20 ± 0.93	19.66	98.29	4.60
1,*N*^6^-εdAdo					
2.08 ± 0.10	0	2.29 ± 0.39	–	–	17.05
2.05 ± 0.04	1	3.06 ± 0.47	1.01	100.89	15.31
1.99 ± 0.06	5	7.87 ± 1.66	5.88	117.60	21.10
2.03 ± 0.07	10	12.43 ± 1.25	10.41	104.06	10.06
1.97 ± 0.03	20	22.42 ± 3.89	20.46	102.29	17.34

### Method validation for 1,*N*^6^-εdAdo and 1,*N*^2^-εdGuo quantification in DNA samples

The calibration curves presented in Additional file 1: Figure S2 show that the MS response was linear in the 1–40 fmol range. Good agreement was also observed between added and detected amounts of 1,*N*^6^-εdAdo and 1,*N*^2^-εdGuo (93–101% accuracy for 1,*N*^2^-εdGuo, and 100–118% accuracy for 1,*N*^6^-εdAdo) (Table 5). Intra-day precision was determined by calculating the coefficient of variation (%) for each concentration. The inter-day precision calculated for DNA aliquots supplemented with 10 fmol of 1,*N*^2^-εdGuo and 1 fmol of 1,*N*^6^-εdAdo was 14.01 and 16.66%, respectively. The limits of on-column quantification (S/N = 10) were 0.3 fmol for 1,*N*^6^-εdAdo and 1 fmol for 1,*N*^2^-εdGuo.

## Discussion

A complex mixture of gases, vapors and particles of varying size and chemical composition is found in urban air, resultant from diverse polluting human activities [6, 17]. To study the effects of specific components of this complex mixture, taking into account the dynamics of pollutants in real ambient air, a feasible approach is to selectively concentrate the desired component over a background of real pollution [75]. Using this approach, we found here that mice exposed to PM_2.5_ from a busy traffic crossroad in São Paulo City presented altered antioxidant system activity in lung, liver, and erythrocytes, concomitantly to increased levels of lung and kidney 8-oxodGuo, kidney 1,*N*^6^-εdAdo, and liver 1,*N*^2^-εdGuo, as well as decreased levels of lung and liver 5-hmC.

In the present study, mice exposed to ambient air enriched in PM_2.5_ (682 ± 532 μg/m^3^) for 1 h daily for 3 months (from infancy to adulthood) were compared to a control group exposed to ambient air, and both groups were treated inside the chambers of a Harvard Ambient Fine-Particle Concentrator located near the crossroad. The total intake dose (ID) of 70.2 μg (1.08 μg per day of exposure) was estimated according to Alexander et al. [85], with the inhalability of the particles determined according to Menache et al. [86] [ID = C × f × VT × I (d50%, ϭg) × t, where C is the PM_2.5_ concentration (600 μg/m^3^), f is the breathing frequency (250 inhalations/min), VT is the tidal volume (0.15 mL), I is the inhalability (d50%, ϭg = 80%), and t is the duration of exposure (3900 min = 65 days, 5 days per week, 1 h per day)]. If mice were exposed to 20 μg/m^3^ PM_2.5_ for 24 h, then the estimated intake dose of particles would be 0.864 μg/day (56.16 μg after 65 days). Thus, we can assume that the adopted exposure protocol mimics a 24 h exposure to concentrations of ambient fine particulate matter found in São Paulo City.

The air pollutants data obtained from the Cerqueira César monitoring station of the São Paulo State Environmental Agency (CETESB, São Paulo, Brazil) for the exposure period of the present study (September 1 to November 30, 2010) showed that the ambient air PM_10_ and PM_2.5_ average (± SD) concentrations were 31.61 ± 22.16 μg/m^3^ and 20.36 ± 11.82 μg/m^3^, respectively [87]. The 2008–2010 triennium average (± SD) PM_10_ and PM_2.5_ concentrations in the Cerqueira César monitoring station were 30.86 ± 5.86 μg/m^3^ and 17.73 ± 1.69 μg/m^3^, respectively [87]. Previous characterization of PM_2.5_ collected in the vicinity of the site of exposure showed that approximately 67% of the PM_2.5_ mass was traffic related [76, 88]. Elemental analysis of PM_2.5_ collected inside the concentrated PM_2.5_ exposure chamber also reinforces that vehicular traffic was the major source of PM_2.5_ in the present study (Table 1), as indicated by the presence of Ca, Mg, Zn, and Pb, which are mainly derived from the combustion of gasoline and oil additives; Zn, Pb, Cd and V, which are derived from diesel tailpipe emissions; and Sb, Fe, Ba, Cu and Al, which are derived from break wear [89]. Polycyclic aromatic hydrocarbons were also detected in PM_2.5_ collected at the site of exposure (Table 2).

The metal content and organic components of PM_2.5_ are important factors for the induction of DNA damage [90, 91]. By direct or indirect mechanisms, both groups of components may induce ROS formation, which, if not equilibrated by cell antioxidant systems, subsequently leads to an increased rate of generation of oxidatively damaged biomolecules [90–96]. A major DNA damage used as a marker of oxidative stress is 8-oxodGuo, a mutagenic lesion formed by the mono electronic oxidation of guanine, or by hydroxyl radical or singlet oxygen attack of guanine in DNA [97].

As reviewed by Møller and coworkers [98], there are few reports on the assessment of oxidatively damaged DNA in the lungs of animals exposed to air pollution particles, with little evidence for associations [98]. Typically, positive associations have been found after rodent exposure to high DEPs concentrations or doses (3 to 80 mg/m^3^, exposure by inhalation for few hours or 1 to 12 months; 0.1 to 4 mg by intratracheal instillation, analysis after a few hours or days) [98, 99]. The long-term inhalation of light-duty diesel engine exhaust (DEPs 3.5 mg/m^3^, 17 h/day, 3 days/week, for 1 to 12 months) led to the induction of 8-oxodGuo in rat lungs at a rate that was higher during the first 3 months of exposure, despite the continuous linear increase in the lung burden of deposited particles along the 12 months [100]. However, the short-term inhalation of a NIST standard reference material 1650 DEP (5 or 20 mg/m^3^, 1.5 h/day, 4 days) did not change the levels of 8-oxodGuo in mouse lungs, which were increased only after a single 1.5 h exposure to 80 mg/m^3^ DEP [101]. Contrary to wild-type mice, repair-deficient *Ogg1*^−/−^ mice exposed by inhalation to the NIST standard reference material 2975 DEP (20 mg/m^3^, 1.5 h/day, 4 days) presented increased lung levels of 8-oxodGuo [98]. Indeed, when the mice were exposed to DEP obtained from a diesel engine (0.05, 0.1 and 0.2 mg) for 10 weeks by weekly intratracheal administration, lung 8-oxodGuo levels were increased (for DEP 0.1 and 0.2 mg) and correlated with lung tumor incidence (for DEP 0.05 and 0.1 mg) [29].

Here, we showed that mice exposed for 3 months to ambient PM_2.5_ at a concentration that leads to the estimated total intake dose of 70.2 μg had increased levels of 8-oxodGuo in the lung tissue (Table 4). This is the first time that such an increase has been experimentally demonstrated for an estimated lung burden of particles close to what would be expected after 24 h/day exposure along 3 months to the average PM_2.5_ concentration found in the local ambient air.

Reported basal levels of 8-oxodGuo in rodent lung tissue, based on HPLC analyses, range from 180 to 450/10^8^ dGuo [44, 101–104], 1340 – 2120/10^8^ dGuo [100], or approximately 3000/10^8^ dGuo [29, 105], with the lowest values obtained from DNA extraction methods by using sodium iodide. The mean 8-oxodGuo level found here in the lung of mice exposed to ambient air was 2124/10^8^ dGuo. The level increased to 2466/10^8^ dGuo in the animals exposed to ambient air enriched in PM_2.5_ (Table 4).

An inter-laboratory assessment of 8-oxodGuo in DNA extracted from standard samples of pig liver and distributed by the European Standards Committee on Oxidative DNA Damage (ESCODD) to different research groups revealed a great discrepancy in the levels detected between laboratories, ranging from 223 to 44,100/10^8^ dGuo, with a median level of 1047/10^8^ dGuo [106]. In the present study, the mean 8-oxodGuo levels found in ambient air exposed mice lung, kidney, and liver DNA were, respectively, 2.0, 1.8, and 2.7 times higher than the median basal level obtained by ESCODD. Considering the limitations of 8-oxodGuo analyses, the DNA samples were extracted and stored by using solutions containing deferoxamine for protection against artifactual oxidation, and the hydrolysis procedure was performed by using 80 μg of DNA from each sample to minimize the contribution of spurious oxidation to the final result, as suggested by Helbock and coworkers [107].

It has been shown that human exposure to each 10 μg PM_2.5_/m^3^ could result in an 11% increase in the levels of 8-oxodGuo in lymphocyte DNA [92]. Increased levels of urinary or serum 8-oxodGuo in humans were also associated with PM_2.5_ mass, its organic and/or elemental constituents, or the duration of exposure [91, 108–110]. Additionally, children exposed to high levels of a complex mixture of air pollutants in Mexico City presented higher levels of 8-oxodGuo in nasal biopsies from the posterior inferior turbinate compared to those of controls [111].

Consistent with an expected increased rate of generation of superoxide radical (O_2_^●-^) in the lung tissue of mice exposed to the concentrated ambient PM_2.5_, lung SOD activity was increased in these animals (Table 3). In a previous study, mice intranasally instilled with a suspension of Residual Oil Fly Ashes (0.2 mg/kg), a surrogate for ambient air PM in many studies, and euthanized 1 or 3 h after exposure, presented increased lung oxygen consumption, NADPH oxidase activity, mitochondrial state 3 respiration, nitric oxide production, phospholipid oxidation, and carbonyl content [112]. Lung SOD activity was also increased in the reported model as a response to the amplified generation of O_2_^●-^ by different sources (NADPH oxidase, mitochondria), favoring O_2_^●-^ dismutation to H_2_O_2_ [103]. Catalase, glutathione peroxidases (GPx) and peroxiredoxins (Prxs) reduce H_2_O_2_ to water, decreasing the chance of its reduction to the highly reactive hydroxyl radical (^•^OH) by transition metals (Fe^2+^, Cu^+^), and preventing the occurrence of oxidative damage [113–115]. While catalase directly decomposes H_2_O_2_ to H_2_O and O_2_, the thiol peroxidases GPx and Prxs reduce H_2_O_2_ to H_2_O via simultaneous oxidation of glutathione (GSH) and thioredoxin (Trx). Oxidized glutathione and thioredoxin (GSSG e TrxS_2_) are reduced by the NADPH-consuming enzymes glutathione reductase (GR) and thioredoxin reductase [113, 115, 116]. Here, the animals exposed to concentrated ambient PM_2.5_ had decreased GR activity in the lung (Table 3), which may unbalance an important defense system against the excess H_2_O_2_ from O_2_^●-^ dismutation, enabling the increased generation of oxidative damage, as observed for 8-oxodGuo. The erythrocytes of the animals exposed to concentrated ambient PM_2.5_ also presented increased SOD activity. This finding is consistent with the knowledge that particles deposited in the lungs not only provoke lung inflammation but also induce a systemic inflammatory response, with the activation of NADPH oxidase in the systemic circulation leading to augmented O_2_^●-^ generation [117].

Exposure to concentrated PM_2.5_ also led to increased levels of DNA lesions in internal organs, such as the liver (augmented 1,*N*^2^-εdGuo) and kidneys (augmented 8-oxodGuo and 1,*N*^6^-εdAdo). As the animals were exposed whole body to the pollutants, the different routes of absorption (pulmonary, gastrointestinal, and dermal) may contribute to these effects. The lesions 1,*N*^2^-εdGuo and 1,*N*^6^-εdAdo are formed by reactive carbonyls resulting from lipid peroxidation [32] and were demonstrated to play a role in the pathophysiology of inflammation [36, 37]. Rats exposed orally to carbon black (0.64 mg/kg b.w.) presented increased levels of 8-oxodGuo, 1,*N*^6^-εdAdo, and 1,*N*^2^-εdGuo in the liver, with the highest effect observed for 1,*N*^2^-εdGuo [44]. However, only 1,*N*^2^-εdGuo levels were increased in the livers of the animals exposed orally to PM (0.64 mg/kg b.w.) collected from a wood stove-rich area [44]. Thus, different profiles of DNA lesions may be induced in response to the exposure of different tissues to different pollutants. A more comprehensive approach quantifying a large panel of lesions will likely reveal yet unknown effects. The differences in these profiles may be influenced by the time the sample was collected, and the inflammatory condition, pollutant biotransformation, and repair capacity of the target tissues [44]. Decreased SOD and increased GST activities were observed in the livers of mice exposed to concentrated PM_2.5_ in the present study, showing a shift of the detoxification system towards the conjugation of reactive electrophiles with glutathione, consistent with the observed increased levels of 1,*N*^2^-εdGuo. The present study is the first to show the induction of etheno adducts in the liver and kidneys of mice exposed to ambient PM_2.5_.

The levels of 1,*N*^6^-εdAdo detected in the present study fall within the range obtained in studies employing ultrasensitive immunoaffinity/^32^P-postlabeling and are lower than those described by other groups employing HPLC-ESI-MS/MS (Table 6). Similarly, the 1,*N*^2^-εdGuo levels quantified in the present study are consistent with the lowest levels reported by Garcia [118] and Angeli [119] by using HPLC-ESI-MS/MS (Table 6).

**Table 6:** Levels of 1,*N*^2^-εdGuo and 1,*N*^6^-εdAdo in DNA from biological samples in different studies

Species/Model	Tissue	Method	Level	References
1,*N*^2^-εdGuo				
Human cell line IMR-90	Lung	LC-ESI-MS/MS	0.2–1.8/10^7^ dGuo	[118]
Human cell line SW480	Colon adenocarcinoma	LC-ESI-MS/MS	0.5–2/10^7^ dGuo	[119]
Human cell line A549	Lung	LC-ESI-MS/MS	1.5–5/10^6^ dGuo	[45]
Rat	Liver	LC-ESI-MS/MS	1–3.25/10^6^ dGuo	[44]
Rat	Lung	LC-ESI-MS/MS	1.5–2.5/10^6^ dGuo	[44]
1,*N*^6^-εdAdo				
Human	Lung	^32^P-postlabeling	2.4–146/10^9^ dAdo	[139]
Human cell line A549	Lung	LC-ESI-MS/MS	0.1–0.45/10^6^ dGuo	[45]
Human	White Blood Cells	^32^P-postlabeling	0.08–24.07/10^8^ dAdo	[140]
Human	White Blood Cells	^32^P-postlabeling	138–1017/10^9^ dAdo	[141]
Human	White Blood Cells	^32^P-postlabeling	0.03–90.15/10^7^ dAdo	[142]
Rat	Liver	LC-ESI-MS/MS	0.75–2/10^6^ dGuo	[44]
Rat	Liver	LC-ESI-MS/MS	2.1–6.9/10^8^ tdn	[143]
Rat	Lung	LC-ESI-MS/MS	0.5–1.25/10^6^ dGuo	[44]

*tdn* total deoxynucleosides

In addition to the genotoxic effect of PM, another important consequence of the exposure is the induction of epigenetic changes, which may affect gene expression without altering the nucleotide sequence in DNA [120–122]. Approximately 70% of the CpG dinucleotides in DNA are methylated, whereas non methylated CpGs are primarily found in “CpG islands”. The dense methylation of promoter regions is associated with gene silencing [123–128].

Here, we observed that PM_2.5_ exposure led to decreased global levels of 5-hmC in lung (34% decrease) and liver (16% decrease) DNA, without affecting the global levels of 5-mC (Table 4). This observation points to global 5-hmC as a more sensitive biomarker. Oxidative stress is a possible contributing factor to the decreased 5-hmC levels. The effect of oxidative stress decreasing the global levels of 5-hmC and modulating the genomic hydroxymethylation profile was demonstrated by Delatte and coworkers [129] in SY5Y human neuroblastoma cell line exposed to buthionine sulfoximine, and in colon epithelia of mice depleted for *GPx*1 and *GPx*2, both situations leading to increased levels of peroxides. We observed a significant inverse correlation between 5-hmC and 8-oxodGuo in mice lung DNA (Fig. 3a). Additionally, several lines of evidence indicate changes in DNA methylation and hydroxymethylation as key events favoring cancer development due to benzo[*a*]pyrene exposure [27, 130–133]. Benzo[*a*]pyrene was one of the PAHs present in the PM_2.5_ collected at the site of exposure (Table 2). Diminished 5-hmC levels were also observed in human bronchial epithelial cells (HBECs) exposed to DEPs (5 mg/cm^2^, 24 h) in vitro [69] and in buccal cells of humans exposed to increased concentrations of PM_2.5_ or PM_10_ [65].

As 5-hmC is a first step in the DNA demethylation pathway catalyzed by TET [57], the decrease in its levels may represent decreased TET activity or increased 5-hmC removal from DNA. Previous studies have suggested that the loss of TET activity may lead to the hypermethylation of gene promoter regions, with the consequent deregulation of gene transcription and cell differentiation [128]. Here we observed that the 5-mC/5-hmC ratio increased in lung DNA of the PM_2.5_ exposed mice (Fig. 3b). Differentiated cells in stratified epithelia of several human and mouse organs presented higher levels of 5-hmC than the stem/progenitor cells and cancer cells [134]. The loss of 5-hmC was suggested to be an early event in carcinogenesis [134].

Ding and coworkers [66] evaluated for the first time the effects of traffic-related air pollution on DNA methylation in rat lung. The authors found, using multiple linear regression, that exposure to PM_2.5_, PM_10_ and NO_2_ was associated with changes in DNA methylation (decreased methylation of LINE1 and *iNOS* promoter, and increased promoter methylation of *APC* and *p16*^*CDKN2A*^) [66]. However, the different exposure localities chosen for the control and exposed groups prevented them from ruling out “the effects from the stress induced by other factors” [66]. In the present study, we focused on the effects of PM_2.5_ concentrated from the ambient air, compared to the ambient air exposed group submitted, in the same place and at the same time, to equal conditions of manipulation and stress, except the concentration of PM_2.5_. This is the first assessment of the levels of DNA lesions, 5-mC, 5-hmC, and oxidative stress in different tissues of mice in such an experimental condition, allowing direct evidence of the effects of PM_2.5_ as it occurs in the environment. However, some short-comings of using the ambient air exposed group as the control must be addressed. The endpoints quantified in this group are not the basal levels that could be present in an unexposed control group. Studies using a clean air site or HEPA filtration in situ to prevent any exposure of the control group to PM would improve the sensitivity for detection of differences between groups.

The assessment of the total levels of 5-mC and 5-hmC in DNA is usually a first step in approaches to understand the effects of different agents on the epigenome, as pointed out by Bakulski and Fallin [52]. Although the total 5-mC and 5-hmC levels in DNA do not give information about the genes affected, this type of analysis provides useful data for the direction of greater resolution studies. For example, (1) detecting changes using the whole DNA denotes a broad effect, which probably encompasses different genes; (2) if the investigated agents affect the epigenetic machinery (e.g., TETs, DNMTs, pathways required to recycle the co-substrates of these enzymes, etc.), changes of the total 5-mC and/or 5-hmC levels are expected [69, 121, 133, 135–137]; (3) a broad effect is more likely to replicate in different animal species, compared to specific changes in genes that depend on different factors, including host development and genetics [121, 129, 133]. On the other hand, if no change is observed in the total levels of 5-mC or 5-hmC, biologically important changes across the genome cannot be excluded. The gain and loss of 5-mC or 5-hmC in different genomic locations may be counter-balanced, resulting in no net change of the global levels, which must be interpreted with caution [135].

Jiang and coworkers [133] assessed the DNA methylation profiles of immortalized human bronchial epithelial cells and murine skin exposed to benzo[*a*]pyrene in vitro and in vivo, respectively. It was evident the broad effect of benzo[*a*]pyrene modulating the DNA methylation in both systems (2414 differentially methylated regions in the exposed murine skin; 105,958 and 20,577 differentially methylated sites in two exposed human cell lines). A total of 153 genes with altered promoter methylation (hypermethylation or hypomethylation) were found in the exposed murine skin, 45 of them (29%) occurred in the exposed human cells. In contrast, 10,447 genes with altered promoter methylation were detected in the exposed human cells [133]. Delatte and coworkers [129] provided the opportunity to compare differentially hydroxymethylated genes induced by oxidative stress in SY5Y human neuroblastoma cell line and in colon epithelia of mice. The global levels of 5-hmC were decreased in both systems under oxidative stress, but the genes and pathways altered, although involved in the oxidative stress response, differed between the systems [129]. Studies comparing the specific agent-induced changes in DNA methylation and hydroxymethylation profiles in tissues of animal models, human cells and human tissues would allow a better understanding of the applicability of such detailed information obtained in an animal model.

LINE1 and Alu repeat elements have been used as surrogate assays for quantifying total 5-mC in the majority of the studies on associations between ambient PM exposure and global DNA methylation alterations [47, 49, 58, 59, 61, 62, 66, 67]. Other methods used for quantification of 5-mC and/or 5-hmC in studies of PM exposure were ELISA [68, 69], indirect assays [63], HPLC-UV [51], HPLC-ESI-MS/MS [64, 65]. LINE1 and Alu assays do not distinguish between 5-mC and 5-hmC, and limit the assessment of methylation levels to specific cytosines in bisulfite converted DNA, quantified by pyrosequencing after polymerase chain reaction [135]. Conversely, HPLC based methods allow the direct quantification of the total levels of DNA bases, including 5-mC and 5-hmC separately, and are then considered the “gold standard” for this purpose [135]. A community-wide benchmarking study compared several methods for DNA methylation analysis [136]. Among the six global DNA methylation assays included in the study (HPLC-MS, ELISA, bisulfite pyrosequencing of four repetitive elements), HPLC-MS data most accurately reflected the expected differences between samples [136].

Using HPLC based methods, De Prins and coworkers [51] found that increased exposure to NO_2_, PM_10_, PM_2.5_ and O_3_ was associated with decreased total 5-mC levels in human blood DNA; Janssen and coworkers [64] observed that total 5-mC levels in human placenta were inversely associated with PM_2.5_ exposure; and Nys and coworkers [65] were the first to show an association between increased human exposure to PM_2.5_ and PM_10_ and decreased 5-mC and 5-hmC total levels in human buccal cell DNA. So far, to the best of our knowledge, this study is the first to quantify 5-mC and 5-hmC by HPLC-ESI-MS/MS in different tissues (lung, liver and kidney) of mice selectively exposed to ambient PM_2.5_. Our observations encourage further investigation of the effects of PM_2.5_ exposure on the profiles of the genomic methylation and hydroxymethylation of target tissues to obtain a better understanding of the deregulated molecular pathways that may culminate in disease (e.g., cancer, metabolic disorders, cardiovascular disease) development [138].

## Conclusions

Mice exposed daily, for 3 months, to PM_2.5_ at a concentration that mimics 24-h exposure to the mean concentration found in ambient air, presented altered antioxidant system activity in lung, liver, and erythrocytes, increased levels of DNA lesions related to oxidative stress in lung, liver, and kidney, and decreased global levels of 5-hmC in lung and liver DNA. This is the first direct demonstration of the occurrence of these effects due to exposure to PM_2.5_ as it occurs in the environment. Genetic and epigenetic alterations induced by pollutants may affect the genes committed to cell cycle control, apoptosis, and cell differentiation, increasing the chance of cancer development, which merits further investigation.

## Additional file

Additional file 1: **Table S1.** Reagents used in the study. **Table S2.** Parameters used in the ESI-MS/MS equipment for detection of the lesions and epigenetic marks in DNA. **Figure S1.** System of two columns used for 8-oxo-7,8-dihydro-2′-deoxyguanosine (8-oxodGuo) analyses. A) Configuration used in the first 16 min and from 32 to 46 min of the chromatography; B) Configuration used in the interval 16 – 32 min, allowing further separation and peak narrowing in column B prior to elution to the ESI source of the mass spectrometer. **Figure S2.** Calibration curves obtained by HPLC-ESI-MS/MS for quantification of 8-oxo-7,8-dihydro-2′-deoxyguanosine (8-oxodGuo), 1,*N*^2^-etheno-2′-deoxyguanosine (1,*N*^2^-εdGuo), 1,*N*^6^-etheno-2′-deoxyadenosine (1,*N*^6^-εdAdo), 2′-deoxycytidine (dCyd), 5-methyl-2′-deoxycytidine (5-mC), and 5-hydroxymethyl-2′-deoxycytidine (5-mC, lower range). (DOCX 738 kb)

## Abbreviations

1,*N*^2^-εdGuo: 1,*N*^2^-etheno-2′-deoxyguanosine
1,*N*^6^-εdAdo: 1,*N*^6^-etheno-2′-deoxyadenosine
5-hmC: 5-hydroxymethylcytosine
5-mC: 5-methylcytosine
8-oxodGuo: 8-oxo-7,8-dihydro-2′-deoxyguanosine
8-oxoGua: 8-oxo-7,8-dihydro-guanine
*APC*: Adenomatous polyposis coli
CAT: Catalase
CDNB: 1-chloro-2,4-dinitrobenzene
CETESB: São Paulo State Environmental Agency
dAdo: 2′-deoxyadenosine
dCyd: 2′-deoxycytidine
DEP: Diesel exhaust particles
dGuo: 2′-deoxyguanosine
DNMT: DNA methyltransferase
ESCODD: European Standards Committee on Oxidative DNA Damage
ESI: Electrospray ionization
GPx: Glutathione peroxidase
GR: Glutathione reductase
GSH: Reduced glutathione
GSSG: Oxidized glutathione
GST: Glutathione S-transferase
HBECs: Human bronchial epithelial cells
HPLC: High performance liquid chromatography
IARC: International Agency for Research on Cancer
*iNOS*: Inducible nitric oxide synthase
LINE1: Long interspersed nucleotide element
LOD: Limit of detection
LOQ: Limit of quantitation
*m/z*: mass-to-charge ratio
MRM: Multiple reaction monitoring
MS: Mass spectrometry
NADPH: Nicotinamide adenine dinucleotide phosphate
NIST: National Institute of Standards and Technology
Ogg1: 8-oxoguanine DNA glycosylase
PAH: Polycyclic aromatic hydrocarbon
PDA: Photodiode array detector
PM: Particulate matter
Prxs: Peroxiredoxins
ROS: Reactive oxygen species
S/N: Signal-to-noise ratio
SD: Standard deviation
SEM: Standard error of the mean
SOD: Superoxide dismutase
TET: Ten-eleven-translocation
Trx: Thioredoxin
