# Supplementary material for: Genotoxic and epigenotoxic effects in mice exposed to concentrated ambient fine particulate matter (PM2.5) from São Paulo city, Brazil
Source: Part Fibre Toxicol. 2018 Oct 19;15:40. doi: 10.1186/s12989-018-0276-y (PMC6194750; doi:10.1186/s12989-018-0276-y)
Supplement: Supplementary file 1 — Table S1. Reagents used in the study. Table S2. Parameters used in the ESI-MS/MS equipment for detection of the lesions and epigenetic marks in DNA. Figure S1. System of two columns used for 8-oxo-7,8-dihydro-2′-deoxyguanosine (8-oxodGuo) analyses. A) Configuration used in the first 16 min and from 32 to 46 min of the chromatography; B) Configuration used in the interval 16 – 32 min, allowing further separation and peak narrowing in column B prior to elution to the ESI source of the mass spectrometer. Figure S2. Calibration curves obtained by HPLC-ESI-MS/MS for quantification of 8-oxo-7,8-dihydro-2′-deoxyguanosine (8-oxodGuo), 1,N2-etheno-2′-deoxyguanosine (1,N2-εdGuo), 1,N6-etheno-2′-deoxyadenosine (1,N6-εdAdo), 2′-deoxycytidine (dCyd), 5-methyl-2′-deoxycytidine (5-mC), and 5-hydroxymethyl-2′-deoxycytidine (5-mC, lower range). (DOCX 738 kb) [file 12989_2018_276_MOESM1_ESM.docx]

**Additional file 1**

**Genotoxic and epigenotoxic Effects in mice exposed to Concentrated Ambient Fine Particulate matter (PM_2.5_) from São Paulo City, Brazil**

Antonio Anax Falcão de Oliveira^† ¥^, Tiago Franco de Oliveira^† ¥ #^, Michelle Francini Dias^†^, Marisa Helena Gennari de Medeiros^‡^, Paolo Di Mascio^‡^, Mariana Veras^§^, Miriam Lemos^§^, Tania Marcourakis^†^, Paulo Hilário Nascimento Saldiva^§ ∞^, and Ana Paula de Melo Loureiro^†^ *

^†^Departamento de Análises Clínicas e Toxicológicas, Faculdade de Ciências Farmacêuticas, Universidade de São Paulo, Av. Prof. Lineu Prestes 580, Bloco 13 B, CEP 05508-000, São Paulo, Brazil.

^‡^Departamento de Bioquímica, Instituto de Química, Universidade de São Paulo, Av. Prof. Lineu Prestes 748, CEP 05508-000, São Paulo, Brazil.

^§^Laboratório de Poluição Atmosférica Experimental – LIM05, Hospital das Clínicas, Faculdade de Medicina, Universidade de São Paulo, Av. Dr. Arnaldo 455, CEP 01246903, São Paulo, Brazil.

^∞^Instituto de Estudos Avançados, Universidade de São Paulo, R. do Anfiteatro, 513 - CEP 05508060, São Paulo, Brazil.

Running title: Genotoxic and epigenetic changes by PM_2.5_

e-mail addresses: [anax.antonio@gmail.com](mailto:anax.antonio@gmail.com), [oliveira@ufcspa.edu.br](mailto:oliveira@ufcspa.edu.br), [michellefrancini5@hotmail.com](mailto:michellefrancini5@hotmail.com), [mhgdmede@iq.usp.br](mailto:mhgdmede@iq.usp.br), [pdmascio@iq.usp.br](mailto:pdmascio@iq.usp.br), [verasine@usp.br](mailto:verasine@usp.br), [mirlemos@usp.br](mailto:mirlemos@usp.br), [tmarcour@usp.br](mailto:tmarcour@usp.br), [pepino@usp.br](mailto:pepino@usp.br), apmlou@usp.br

^¥^Contributed equally to this work

^#^Current address of T.F.O.: Departamento de Farmacociências, Universidade Federal de Ciências da Saúde de Porto Alegre, Rua Sarmento Leite 245, CEP 90050-170, Porto Alegre, Rio Grande do Sul, Brazil.

*Corresponding author: Phone: ++(55)1130911192, e-mail: [apmlou@usp.br](mailto:apmlou@usp.br)

**Table S1.** Reagents used in the study.

| **Product** | **Manufacturer** | **Catalogue number** |
| --- | --- | --- |
| [^15^N_5_]-2’-deoxyadenosine | Cambridge Isotope Laboratories | NLM-3895-25 |
| [^15^N_5_]-2’-deoxyguanosine | Cambridge Isotope Laboratories | NLM-3899-CA-10 |
| 1-chloro-2,4-dinitrobenzene | Aldrich | 237329 |
| 2’-deoxycytidine | Sigma | D3897 |
| acetonitrile | Carlo Erba Reagents | 412413000 |
| alkaline phosphatase from bovine intestinal mucosa | Sigma | P5521 |
| ammonium acetate | Merck | 101116 |
| calf thymus DNA | Sigma | D1501 |
| cell lysis solution | QIAGEN | 158908 |
| chloroform | Carlo Erba Reagents | 412653 |
| deferoxamine | Sigma | D9533 |
| DNase I | Bio Basic Inc | DD0649 |
| EDTA | Sigma | E5391 |
| ethanol | Carlo Erba Reagents | 414542 |
| formic acid | Sigma-Aldrich | F0507 |
| hydrochloric acid | Carlo Erba Reagents | 302601 |
| hypoxanthine | Sigma | H9377 |
| isoamyl alcohol | Sigma-Aldrich | M32658 |
| isopropyl alcohol | Carlo Erba Reagents | A412790010 |
| L-glutathione oxidized | Sigma | G6654 |
| L-glutathione reduced | Sigma-Aldrich | G4251 |
| L-glutathione reductase | Sigma | G3664 |
| magnesium chloride | Carlo Erba Reagents | 349377 |
| magnesium chloride | Sigma | M2393 |
| methanol | Carlo Erba Reagents | L022909K7 |
| NADPH | Sigma | N1630 |
| phosphodiesterase I from *Crotalus atrox* | Sigma | P4506 |
| potassium phosphate dibasic | Sigma-Aldrich | P3786 |
| potassium phosphate monobasic | Merck | 104873 |
| potassium phosphate monobasic | Sigma-Aldrich | P5379 |
| protein precipitation solution | QIAGEN | 158912 |
| proteinase K | Sigma-Aldrich | P2308 |
| ribonuclease A | Sigma | R5000 |
| sodium chloride | Sigma-Aldrich | S9625 |
| sodium hydroxide | Merck | 106498 |
| tert-butyl hydroperoxide | Sigma-Aldrich | B2633 |
| tris(hydroxymethyl)-aminomethane | Carlo Erba Reagents | 489983 |
| xanthine oxidase | Sigma | X4500 |

**Table S2.** Parameters used in the ESI-MS/MS equipment for detection of the lesions and epigenetic marks in DNA.

| **ESI-MS/MS parameters** |  | **8-oxodGuo** |  | **Etheno adducts** |  | **5-mC and 5-hmC** |
| --- | --- | --- | --- | --- | --- | --- |
| *Curtain Gas* |  | 20 psi |  | 20 psi |  | 15 psi |
| *Nebulization Gas* |  | 55 |  | 50 |  | 45 psi |
| *Ion Source Gas* |  | 50 psi |  | 40 psi |  | 50 psi |
| *Collision-induced Dissociation Gas* |  | Medium |  | Medium |  | Low |
| *Ion Spray Voltage* |  | 5000 |  | 4500 |  | 5500 |
| *ESI Probe Temperature* |  | 450 |  | 450 |  | 600 |
| *Declustering Potential* |  | 31 V, 8-oxodGuo |  | 41 V, 1,*N*^6^-εdAdo |  | 36 V, dCyd |
|  |  | 31 V, [^15^N_5_]8-oxodGuo |  | 41 V, [^15^N_5_]1,*N*^6^-εdAdo |  | 36 V, 5-mC |
|  |  |  |  | 45 V, 1,*N*^2^-εdGuo |  | 36 V, 5-hmC |
|  |  |  |  | 45V, [^15^N_5_]1,*N*^2^-εdGuo |  | 41 V, [^15^N_5_]1,*N*^6^-εdAdo |
| *Collision Energy* |  | 23 eV, 8-oxodGuo |  | 25 eV, 1,*N*^6^-εdAdo |  | 15 eV, dCyd |
|  |  | 23 eV, [^15^N_5_]8-oxodGuo |  | 25 eV, [^15^N_5_]1,*N*^6^-εdAdo |  | 15 eV, 5-mC |
|  |  |  |  | 27 eV, 1,*N*^2^-εdGuo |  | 15 eV, 5-hmC |
|  |  |  |  | 27 eV, [^15^N_5_]1,*N*^2^-εdGuo |  | 25 eV, [^15^N_5_]1,*N*^6^-εdAdo |
| *Collision Cell Exit Potential* |  | 16 V, 8-oxodGuo, |  | 8 V, 1,*N*^6^-εdAdo |  | 6 V, dCyd |
|  |  | 16 V, [^15^N_5_]8-oxodGuo |  | 8 V, [^15^N_5_]1,*N*^6^-εdAdo |  | 6 V, 5-mC |
|  |  |  |  | 16 V, 1,*N*^2^-εdGuo |  | 6 V, 5-hmC |
|  |  |  |  | 16 V, [^15^N_5_]1,*N*^2^-εdGuo |  | 8 V, [^15^N_5_]1,*N*^6^-εdAdo |
| *Entrance Potential* |  | 10 V |  | 10 V |  | 10 V |


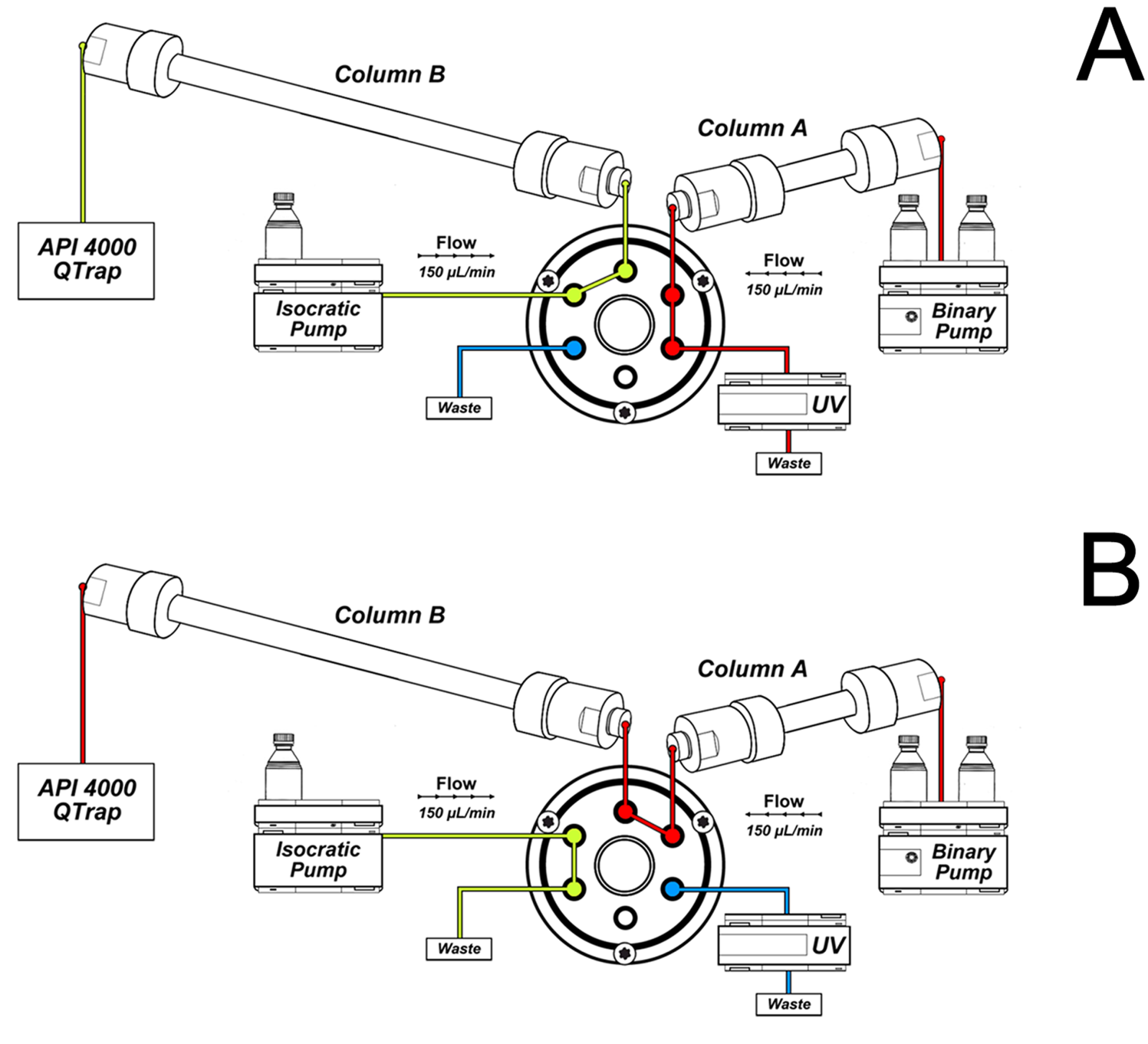


**Figure S1.** System of two columns used for 8-oxo-7,8-dihydro-2’-deoxyguanosine (8-oxodGuo) analyses. A) Configuration used in the first 16 min and from 32 to 46 min of the chromatography; B) Configuration used in the interval 16 – 32 min, allowing further separation and peak narrowing in column B prior to elution to the ESI source of the mass spectrometer.


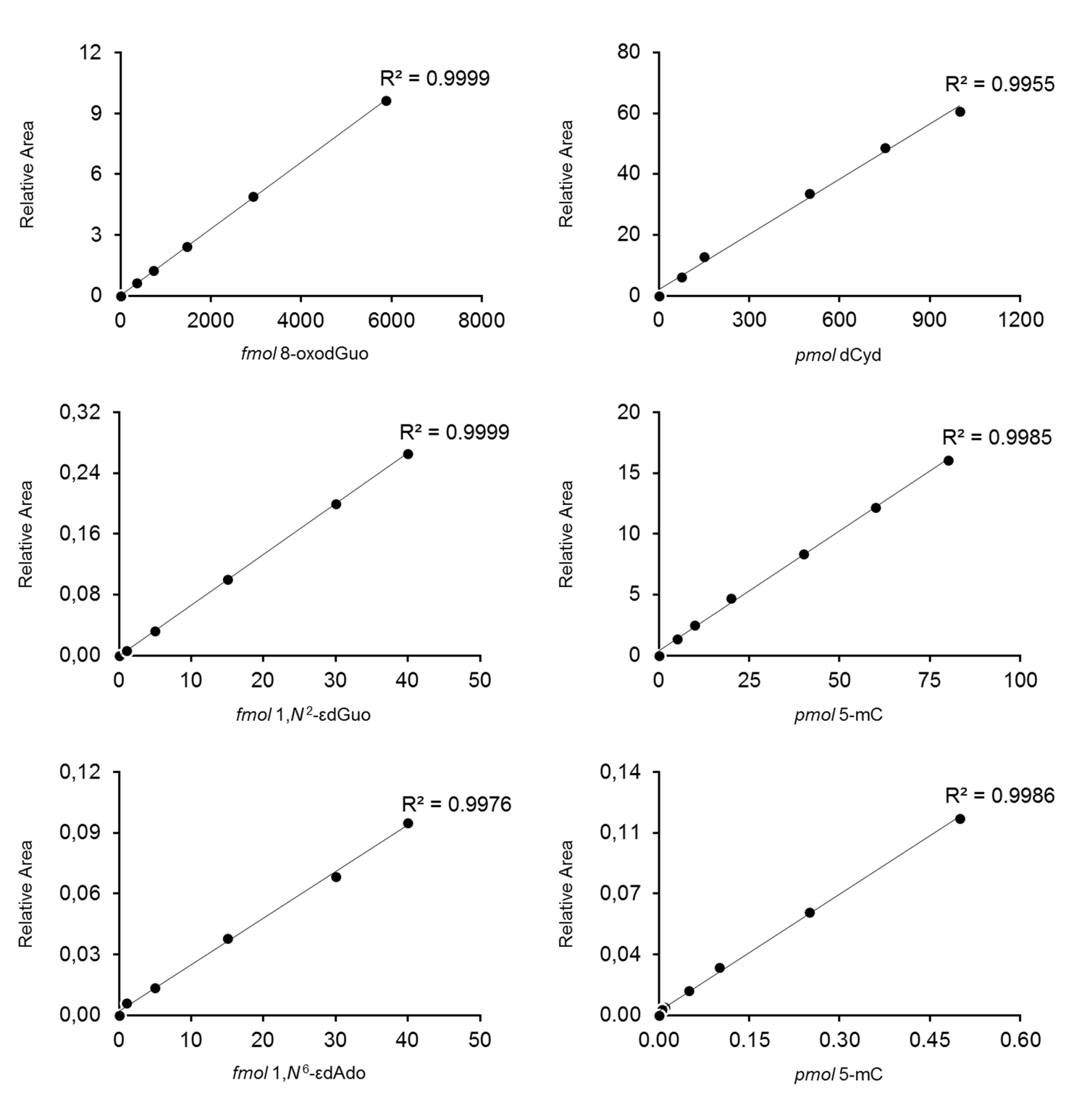


**Figure S2.** Calibration curves obtained by HPLC-ESI-MS/MS for quantification of 8-oxo-7,8-dihydro-2’-deoxyguanosine (8-oxodGuo), 1,*N*^2^-etheno-2´-deoxyguanosine (1,*N*^2^-εdGuo), 1,*N*^6^-etheno-2´-deoxyadenosine (1,*N*^6^-εdAdo), 2´-deoxycytidine (dCyd), 5-methyl-2´-deoxycytidine (5-mC), and 5-hydroxymethyl-2´-deoxycytidine (5-mC, lower range).
